# Membrane trafficking in podocyte health and disease

**DOI:** 10.1007/s00467-012-2281-y

**Published:** 2012-08-30

**Authors:** Agnieszka Swiatecka-Urban

**Affiliations:** Department of Nephrology, Children’s Hospital of Pittsburgh, Pittsburgh, PA 15224 USA

**Keywords:** Podocyte, Membrane trafficking, Endocytosis, Recycling, Cytoskeleton, Proteinuria signaling, Glomerular slit diaphragm

## Abstract

Podocytes are highly specialized epithelial cells localized in the kidney glomerulus. The distinct cell signaling events and unique cytoskeletal architecture tailor podocytes to withstand changes in hydrostatic pressure during glomerular filtration. Alteration of glomerular filtration leads to kidney disease and frequently manifests with proteinuria. It has been increasingly recognized that cell signaling and cytoskeletal dynamics are coupled more tightly to membrane trafficking than previously thought. Membrane trafficking coordinates the cross-talk between protein networks and signaling cascades in a spatially and temporally organized fashion and may be viewed as a communication highway between the cell exterior and interior. Membrane trafficking involves transport of cargo from the plasma membrane to the cell interior (i.e., endocytosis) followed by cargo trafficking to lysosomes for degradation or to the plasma membrane for recycling. Yet, recent studies indicate that the conventional classification does not fully reflect the complex and versatile nature of membrane trafficking. While the increasing complexity of elaborate protein scaffolds and signaling cascades is being recognized in podocytes, the role of membrane trafficking is less well understood. This review will focus on the role of membrane trafficking in podocyte health and disease.

## Membrane trafficking

All living cells process information by trafficking cargo, such as extracellular ligands, microorganisms, nutrients, transmembrane proteins and lipids from the plasma membrane to endocytic vesicles (i.e., endocytosis). A reciprocal process called recycling balances endocytosis and returns much of the internalized membrane and cargo to the cell surface. The balance between endocytosis and recycling controls the plasma membrane composition and provides cells with information that has been resolved in time and space. A wealth of new evidence is uncovering the role of endocytosis and recycling as master regulators of diverse cellular functions such as nutrient uptake and metabolism, development, proliferation, differentiation and polarity, reprogramming, migration, cell adhesion and migration, cytokinesis, and neurotransmission [1–3]. Endocytic and recycling pathways are very dynamic and highly coordinated and allow cells to turn over an equivalent of the entire plasma membrane one to five times per hour. Such dynamic organization of membrane trafficking requires diverse protein networks, including the Bin/Amphiphysin/Rvs (BAR) superfamily of proteins, guanine triphosphate hydrolases (GTPases), non-conventional progressive motors, cytoskeletal proteins and their regulators, cell adhesion and sorting molecules, channels, members of the ubiquitin pathway, and lipids [1, 4]. Although endocytosis and recycling are ubiquitous, specific trafficking itineraries are cell- and cargo-specific and depend on the state of cell polarization. Endocytic trafficking can be constitutive and/or ligand activated. Alterations of membrane trafficking can manifest as congenital syndromes and malformations, cancer, inflammation, and immunodeficiency [1].

### Mechanisms of endocytic entry

Endocytosis occurs by various mechanisms that can be broadly divided into clathrin-dependent and clathrin-independent (Table 1). The clathrin-dependent endocytosis (CDE) is one of the most important internalization routes in eukaryotic cells [1]. In CDE, endocytic adaptors recognize linear internalization sequences located in the intracellular C-terminal domains of membrane proteins destined for endocytosis and recruit these proteins as cargo at membrane domains engulfed on the cytosolic side with clathrin lattices called clathrin-coated pits (CCPs). Subsequently, additional adaptors cleave off CCPs at the neck and release them into the cell interior as clathrin-coated vesicles (CCVs). Clathrin-independent endocytosis (CIE) encompasses a diverse group of internalization mechanisms sharing a common requirement for free cholesterol, proteins, and lipids that reside in sphingolipid-rich lipid raft membranes [5]. Collectively, CIE may account for as much as 50 % of the total endocytic activity in a cell (Table 1).

**Table 1:** Mechanisms of endocytosis

Pathway	Morphology and size	Coat	Small GTPase	Cargo	Function
Clathrin-mediated^a^	Vesicular 150–200 nm	Clathrin	Rab5	RTKs, GPCR, TGF-ßR, Notch, TfR, LDLR, ß-arrestin, Wnt/ß-catenin	Cell signaling, vesicular transport
Caveolae-mediated^a^	Flask-shaped 50–120 nm	Caveolin 1 and 2	Unclear	GPI-APs, TGF-ßR, CTxB, viruses, folic acid, IGF-1R, Wnt/ß-catenin	Cell signaling, lipid regulation, vesicular transport, transcytosis
CLIC/GEEC	Tubular	None	Cdc42 Arf1	GPI-APs, glycosphingolipids, cholera toxin	Actin dynamics and cellular stress pathways, differentiation and apoptosis, focal adhesion, fluid-phase uptake, oncogenesis
Arf6-mediated	Tubular	None	Arf6	ß-arrestins, MHC I-II	Membrane curvature
Flotillin-mediated	Vesicular	Flotillin 1 and 2	None	CTxB, GPI-AP, proteoglycans	Lipid raft-mediated endocytosis,
IL-2R^a^	Vesicular 50–100 nm	None	RhoA, Rac1	IL-2Rß, yc cytokine receptor	IL-2R endocytosis and signaling
Macropinocytosis^a^	Ruffled	None	Rac1, Cdc42, Arf6, Rab5	Fluid, RTKs, bacteria	Extracellular fluid uptake, actin dynamics
Phagocytosis	Cargo shaped	None	Rac1, RhoA, Cdc42	Nutrients, pathogens, dead cells, and cellular debris	Uptake of nutrients, pathogens, and cellular debris by professional phagocytic cells; opsonization of foreign particles followed by actin rearrangements

Arf, ADP rybosylation factor; GTPase, guanine triphosphate hydrolyze; Cdc42, cell division control protein 42 homolog; CLIC, clathrin-independent carriers; CTxB, cholera toxin B subunit; GEEC, GPI-AP-enriched early endosomal compartment; GPI-APs, glycosphingolipids anchored proteins; GPCR, G-protein coupled receptor; Rab, ras-associated binding protein; IGF-1R, insulin growth factor-1 receptor; IL-2R, interleukin 2 receptor beta; TRKs, receptor tyrosine kinases; TfR, transferring receptor; LDLR, low-density lipoprotein receptor; MHC, Major histocompatibility complex; RhoA, Ras homolog gene family, member A; Rac1, Ras-related C3 botulinum toxin substrate 1; TGF-ßR, transforming growth factor beta receptor

^a^marks the dynamin-dependent mechanisms; macropinocytosis requires dynamin only in some situations. The above information is based on references listed in this manuscript (most extensively reviewed in refs. [1, 2]. Only selected cargo and functions are listed

### Membrane remodeling and vesicle severing

Remodeling of the plasma membrane at the onset of endocytosis starts with the formation of functional nanoscopic domains and leads to local membrane deformation and curvature change [4]. The BAR domain proteins recognize changes in membrane curvature, facilitate interactions between the plasma membrane and endocytic adaptors, and lead to membrane invagination directed towards the cell interior. Similarly, endophilins A1-3 sense and bend plasma membranes to form CCPs (see below). For most, but not all of the endocytic entry mechanisms, the large GTPase, dynamin cleaves off the budded vesicles at the neck and releases them into the cell interior.

### Endocytic compartments

Regardless of the mechanism of endocytosis, internalized cargo is transported to early endosomes (EE), the focal point of the endocytic pathway. EEs comprise functionally and morphologically heterogenous vesicular membranes consisting of distinct sub-domains such as the multi-vesicular region with large intraluminal vesicles (300–400 nm) and tubular extensions (60–80 nm diameter) [4]. EEs sort internalized cargo to several destinations, either late endosomes (LE) followed by traffic to lysosomes for degradation or the *trans*-Golgi network (TGN) or recycling endosomes (RE) for recycling to the plasma membrane. Several hypotheses have been proposed to explain how cargo is sorted from EEs to subsequent destinations. The prevailing view is that sorting is achieved through recruitment of specific adaptors synchronized with changes in the local pH and clustering of cargo in the EE sub-domains followed by morphological changes in the EE membranes to release the specialized cargo containing vesicles [4]. Cargo targeted for degradation clusters in the multi-vesicular region of EEs where the pH decreases from 6.2 to ~5.5 and is subsequently released into multivesicular bodies (MVB) that become LEs and ultimately lysosomes. By contrast, cargo targeted for recycling clusters in the tubular extensions of EEs where the pH rises to ~6.5.

The EE membranes are characterized by presence of numerous protein adaptors [6]. The small GTPases, Ras-associated binding (Rab) proteins play a critical role in orchestrating cargo sorting. Rabs cycle between the active or GTP bound, membrane-associated state and the inactive or GDP bound, soluble state. The changes in Rab activity are coupled to the reversible association with target membranes. In the active (i.e., membrane bound) state, Rabs recruit diverse effectors to the target vesicles and together control cargo selection, vesicle budding and tethering, and membrane fusion. Rab5 is enriched in EEs.

### Recycling mechanisms

By way of EE tubulation and acquisition of specific adaptors luminal cargo can be recycled back to the plasma membrane via the fast (t1/2 = 5 min) and slow (t1/2 = 15–30 min) recycling pathways [7]. For classic CDE cargo such as the transferrin receptor (TfR) and low-density lipoprotein (LDL) receptor, the fast recycling pathway is considered a default route not requiring sequence motifs for recognition and sorting. During fast recycling, TfR is sorted to the Rab4 and away from the Rab5 microdomain on the same vesicle [8]. Although Rab4 has an established role in the fast recycling pathway, its precise role has not been established as studies demonstrate that expression of the GDP locked, dominant interfering Rab4 mutant inhibits fast recycling while Rab4 depletion increases rapid recycling, perhaps by blocking trafficking via the slow recycling pathway [8]. Recent studies have focused on the role of Rab35 (also known as receptor-mediated endocytosis or RME-5) as an important regulator of fast recycling [9]. Rab35 localizes to the plasma membrane and EEs and is required for fast recycling of TfR. Recruited of Rab35 to CCPs suggests that it plays a role in fast recycling of the CDE cargo. Moreover, association of Rab35 with the ADP rybosylation factor 6 (Arf6)-positive tubular REs suggests that Rab35 may also play a role in fast recycling of the CIE cargo.

Alternatively, cargo, including TfR can be sorted from EEs to the juxtanuclear endocytic recycling compartment (ERC) via the slow recycling pathway. This pathway is usually studied when experimentally measuring recycling and involves trafficking from the EEs to ERC and from the ERC to the plasma membrane. ERC is a tubular compartment devoid of fluid and is defined by presence of either Rab11 or EHD1 (RME-1 family of carboxy-terminal epidermal growth factor receptor substrate 15 (EPS15) homology domain containing protein 1)) or both [10]. EHD1 is scaffolding, membrane tabulating, and possibly membrane fission protein and an important regulator of recycling out of the ERC. Sorting nexins direct cargo from the EEs to ERC while preventing entry into the degradative compartment [11]. Recycling from the ERC to plasma membrane occurs in via two distinct compartments. Although both require Rab11, the REs utilized for TfR trafficking differ from the tubular REs that carry the CIE cargo internalized back to the plasma membrane. The previously held view that cargo lacking the biosynthetic of degradative signals is sorted for recycling in the ERC has been challenged by identification of sorting motifs for recycling, unlike in the fast recycling route, where recycling sequence motifs are considered unnecessary [12].

### Lysosomal targeting

Trafficking of cargo destined for lysosomal degradation requires signals for recruitment of the sorting machinery [13]. Ubiquitination (i.e., attachment of ubiquitin molecules to lysine residues on target proteins) serves as such signal [14]. Ubiquitinated cargo is recognized in EEs by hepatocyte growth factor regulated tyrosine kinase substrate (Hrs) via its ubiquitin interacting motif (UIM). Hrs also interacts with the multi-vesicular region of the EEs involved in cargo sorting for degradation and away from tubule forming, recycling membranes. Two additional UIM containing proteins, epidermal growth factor receptor substrate 15 (Eps15) and signal transducing adaptor molecule 2 (STAM2) stabilize the association of Hrs with the ubiquitinated cargo and form a functional sorting complex. Subsequent binding of Hrs to the tumor susceptibility gene 101 (Tsg101) subunit of the endosomal sorting complex required for transport (ESCRT-I) recruits the ubiquitinated cargo complex to LEs. Assembly of ESCRT complexes leads to formation of MVBs, lysosomal fusion, and cargo degradation.

### Transcytosis

Polarized epithelial cells require protein delivery to apical or basolateral membranes. Many proteins reach their final destination via direct vectorial transport from the TGN. By contrast, other proteins, initially delivered to one membrane domain are subsequently endocytosed and redirected to the ultimate domain via transcytosis [15]. Transcytosis requires post-endocytic entry into a specialized recycling route, in which proteins are destined for recycling to the ultimate membrane domain or transport to lysosomes. Although presence of transcytosis has been well established, its functional importance varies in different cell types and its role in overall epithelial polarity is inconclusive.

### Membrane trafficking and cell signaling

Membrane trafficking coordinates signaling by directing ligands, receptors, and effectors at a precise time to specific microdomains where signaling is required [1, 2]. In this manner, membrane trafficking may initiate or stop and accelerate or attenuate signaling by engaging or disengaging signaling cascades in vesicular compartments. Specifically, membrane trafficking may regulate net signaling output: (a) by controlling accessibility of ligands, receptors, and accessory proteins at the plasma membrane; (b) by targeting ligand-receptor complexes to different endocytic routes; (c) by sorting internalized ligand-receptor complexes for either recycling or degradation; and (d) by recruiting different signaling pathways to the same vesicular compartment to orchestrate cross-talk.

## Podocytes

The kidney glomerulus is a filtering apparatus allowing passage of water and solute into the urinary space while retaining the vast majority of plasma proteins within the circulation. Podocytes together with the glomerular basement membrane (GBM) and fenestrated capillary endothelial cells form a morphological and functional unit called the glomerular filtration barrier (GFB) [16]. Although, the precise mechanism of GFB selectivity is still a matter of debate, the cross-talk between components of the GFB and the subpodocyte space determines glomerular filtration characteristics [16]. The mature podocyte consists of the cell body, the microtubule and intermediate filament-based primary and secondary processes, and actin-based tertiary or foot processes (FP). Resting on the GBM, FPs form interdigitating extensions linked by the glomerular slit diaphragm (GSD), which provides the only cell-cell contact between mature podocytes [17]. The development, differentiation, and maintenance of mature podocytes require temporally and spatially coordinated activation of various protein networks and signaling cascades. Our current understanding of role and mechanisms of membrane trafficking in these aspects of podocyte biology are reviewed below.

### Membrane trafficking during podocyte development and differentiation

Kidney development is a process of morphogenesis and patterning that eventually leads to formation of highly specialized cells tailored to perform unique functions [18]. Four stages characterize glomerulogenesis of mammalian kidneys [18]. During the first stage the renal vesicle appears—the primordial epithelial structure that originates from the cap mesenchyme. During the second stage the comma-shaped body forms, followed by the S-shaped body that subsequently gives rise to the precursors of podocyte and renal tubules. The glomerular capillary system appears during the third stage and glomerular maturation occurs during the fourth stage.

Formation of the renal vesicle from the cap mesenchyme during the first stage of glomerulogenesis is initiated by induction of undifferentiated mesenchyme by signals from the ureteric bud (UB) to undergo mesenchymal-epithelial transition (MET) [19]. An array of factors and receptors mediate MET in a manner highly coordinated in regards to time and space. Wnt/*ß*-catenin and transforming growth factor ß (TGF-ß) signaling pathways regulate MET during glomerulogenesis [20]. The Wingless-type mouse mammary tumor virus integration site family, member 4 (Wnt4) ligand is necessary and sufficient for MET during glomerulogenesis and is regulated by the Wilms’ tumor suppressor-1 (WT1) transcription factor [21]. Wnt ligands bind to frizzled receptor and the LDL 5 or 6 coreceptor to destabilize the ß-catenin destruction complex [2]. The Wnt/*ß*-catenin signaling utilizes two endocytic routes, CDE and caveolae-mediated CIE [2]. While CIE activates, CDE inhibits Wnt/*ß*-catenin signaling [1]. Similarly, the TGF-ß receptor complex (TGF-ßR) exploits both CDE and caveolae-mediated CIE. However, unlike Wnt/*ß*-catenin, TGF-ß signaling is activated by CDE and terminated by CIE (at least in some cases; Table 1). The TGF-ß pathway directs the action of Wnt during MET and it is plausible that both pathways converge in endocytic vesicles during signaling. Mesenchymal induction is also regulated by epidermal growth factor (EGF) and fibroblast growth factor (FGF) receptors and membrane trafficking is critical for their signaling [1].

Podocyte formation occurs during the second stage of glomerulogenesis, when the proximal segment of the S-shaped body differentiates to form the parietal (Bowman capsule) and the visceral (podocytes) epithelium [18]. Notch signaling controls segmentation of the comma-shaped body and generation of the S-shaped body, which further differentiates to form podocytes [22]. Signal transduction is initiated by engagement of the plasma membrane Notch receptors expressed on the signal-receiving cells with ligands anchored at the plasma membrane on signal-sending cells. Membrane trafficking is critical for spatial and temporal regulation of Notch signaling and involves CDE and CIE [1, 2]. CDE facilitates unfolding and proteolysis of the Notch receptor essential for its activation. Moreover, endocytosis and recycling recruit ligands to restricted regions of the plasma membrane and promote high local ligand abundance resulting in robust Notch activation [23]. To date, membrane trafficking has not been studied in Notch signaling during podocyte development. These studies could increase our understanding of temporal and spatial regulation of podocyte development. Moreover, examining the dynamic regulation of Notch signaling by membrane trafficking could resolve some differences observed in the role of Notch in podocyte development [24, 25].

Semaphorin3a (Sema3a) is another signaling molecule recently shown to play a crucial role in podocyte differentiation in vivo where tight regulation of Sema3a signaling is essential for developing normal GFB [26]. The mechanism whereby Sema3a signaling is controlled during glomerulogenesis remains unknown. However, based on recent data, it can be hypothesized that control of Sema3a signaling is mediated by expression of transcription factors that regulate rates of flotillin-mediated CIE of Sema3a receptors [27]. It remains to be determined whether flotillin-mediated CIE coordinates Sema3a signaling during glomerulogenesis.

### Membrane trafficking during podocyte maturation

Epithelial cell maturation involves polarized (i.e., asymmetric) distribution of signaling molecules and cytoskeletal components that subsequently organize growth of distinct cellular structures. Podocyte maturation leads to asymmetric distribution of protein complexes in specialized membrane domains that allow contact between FPs via GSDs and between FPs and the GBM via focal contacts (FC). To acquire polarized podocyte organization, primordial columnar epithelial cells lose some of the typical epithelial characteristics, such as expression of cytokeratin and desmosomal proteins and regain *de novo* some mesenchymal features, such as expression of the intermediate filament protein, vimentin [28]. Ultimately, the mature podocyte become organized as an arborized structure defined by the unique architecture and contractile apparatus. The GSD forms during podocyte maturation when the apical junctional complex (AJC) of primordial columnar epithelium migrates laterally from apex to base. After migration of AJCs the podocyte cell body extends apically towards Bowman’s capsule and FPs develop at the basal pole. Although there is little direct evidence from maturing podocytes, studies in other model systems demonstrate that endocytosis and recycling facilitate migration of AJCs along the lateral membrane by organizing disassembly of the old and assembly of the new junctional complexes [29].

Much insight into the role of membrane trafficking in breaking the symmetry that takes place during cell polarization has been learned from studying budding yeast. It has been demonstrated that three trafficking events are sufficient to achieve and maintain the asymmetric distribution of proteins that initiate and maintain cell polarization [30]. First, proteins are transported in vesicles from the cytoplasm to the plasma membrane along the actin and microtubule filaments referred to as directed transport. Second, proteins inserted into the plasma membrane cluster in micro-domains via two-dimensional diffusion. Third, proteins undergo a turnover between the cell membrane and interior by endocytosis and recycling. The rates and feedbacks from the trafficking events and the cross-talk between trafficking and the actin and microtubule cytoskeleton are sufficient to determine the shape of polarized distribution, the strength of polarization, and ultimately the morphogenic fate [31].

Several protein complexes are known to initiate and maintain cell polarization. The Rho GTPase, cell division control protein 42 homolog (Cdc42) is the prototypical protein required for polarization, recently confirmed to play a critical role in podocyte polarization [32]. The evolutionarily conserved polarity complexes, Crumbs and Scribble control apical and basolateral polarity, respectively and both play a role in podocyte maturation [33]. The tight junction (TJ)-associated partitioning defective (PAR), Par3/Par6/activated protein kinase C (aPKC) polarization complex is essential in establishing and maintaining polarity by facilitating asymmetric targeting of proteins that maintain functional differences between the apical and basolateral membrane [34]. Par3/Par6/aPKC is essential for podocyte polarization and maintenance of GSD function [35]. In response to polarization signals in podocyte precursors, Par3/Par6/aPKC complexes migrate the apicolateral AJCs towards the basolateral location through mechanisms described above, namely directed transport, two-dimensional diffusion and endocytic recycling until assuming the ultimate location of the GSD [36, 37].

### Membrane trafficking at the glomerular slit diaphragm (GSD)

Despite retaining some features of the AJC, namely the TJ and adherence junction (AJ), the mature GSD has unique composition and architecture consisting of membrane bridging protein networks and juxtaposed cytoplasmic protein complexes that interact with the actin cytoskeleton [38]. The GSD maintains selective permeability between the blood and urinary space (gate function), separates the apical and basolateral plasma membrane (fence function), and serves as a signaling platform (signaling function). While ultrastructural observations of the AJC and GSD convey an image of a static structure, overwhelming evidence demonstrates that dynamic remodeling by membrane trafficking is essential for the fence, gate and signaling function [39, 40].

Nephrin, a transmembrane protein critical for the GSD function belongs to the immunoglobulin superfamily of cell adhesion molecules (IgCAM) [41]. The extracellular domains of nephrin molecules expressed between adjacent FPs form homophilic interactions [42]. The dynamic model of the GSD as well as the pathophysiological, morphological, and biochemical changes resulting from absence or mislocalization of nephrin predict that membrane trafficking is vital to nephrin function [43, 44].

Nephrin localization at the GSD and its function is critically dependent on podocin, the integral membrane protein localized at the GSD lipid raft domains [45]. *Podocin* gene mutations associated with steroid resistant hereditary and sporadic nephritic syndrome (NS) lead to nephrin mislocalization because the mutant podocin is absent from the GSD lipid rafts [45, 46]. Podocin belongs to a large family of integral membrane proteins carrying an evolutionary conserved prohibitin homology (PHB) domain—a primordial lipid recognition motif [47]. While the molecular mechanism of podocin-mediated nephrin recruitment to lipid rafts is unknown, studies of other proteins containing the PHB domain, namely flotillins shed some light. Flotillins tightly associate with the inner leaflet of the plasma membrane via the PHB domain located near the N-terminus and via palmitoylated and myristoylated domains upstream of the N-terminus [48]. The PHB domain contains hydrophobic regions capable of forming a hairpin that helps to insert the protein into the plasma membrane inner leaflet. The predicted topology of mouse podocin has intracellular N- and C-terminal domains, a transmembrane domain, and C-terminally located PHB domain that, unlike the PHB of flotillins, is not involved in hairpin formation [45]. Flotillins form homo- and heterophillic interactions mediated via the C-terminal region conserved only within the flotillin family while in podocin both the N- and C-terminal domains were shown to mediate homooligomerization [45, 48]. As of now, nothing is known about the hydrophobic stretches that form the hairpin in podocin and whether podocin interactions with GSD lipid rafts involve palmitoylation and/or myristoylation.

Flotillins orchestrate lipid raft-based signaling platforms and flotillin-rich membranes that are critical for endocytosis of several receptors, some of which require the Src family kinase, Fyn [49]. Nephrin phosphorylation by Fyn augments the interaction with podocin and facilitates nephrin CIE and signaling [50, 51]. Nephrin was implicated to undergo CDE and CIE [51–53]. Utilizing different endocytic itineraries by a single receptor is a common phenomenon and serves to diversify signaling specificity and strength [1]. Presence of several putative tyrosine-based endocytic motifs in the cytoplasmic C-terminal tail of nephrin supports the notion that nephrin may undergo CDE. While lipid raft-mediated CIE is facilitated by nephrin phosphorylation and augments nephrin signaling, CDE could play a role in attenuating nephrin signaling. A prerequisite for CDE would be dissociation of the nephrin–podocin complex followed by nephrin partitioning to non-raft membrane domains. Quack et al. demonstrated that ß-arrestin mediates nephrin endocytosis by a mechanism that requires dephosphorylation of the Y^1193^ residue in the nephrin cytoplasmic tail [54]. By contrast, phosphorylation of the Y^1193^ residue by Fyn augments nephrin interaction with podocin, prevents nephrin interaction with ß-arrestin, and attenuates nephrin endocytosis while augmenting nephrin signaling [54]. The mechanism of ß-arrestin-mediated nephrin internalization in podocytes is not completely understood. ß-arrestins are known to interact predominantly with the CDE adaptors, clathrin and the assembly polypeptide-2 complex (AP-2) but may also interact with Arf6 in the CIE pathway [55, 56]. It can be predicted that phosphorylation of Y^1193^ may inhibit CDE by inactivating the Y^1193^-based endocytic motif in nephrin. Y^1193^ and subsequent amino acid residues D-E-V in human nephrin conform to a canonical, tyrosine-based endocytic signal of the YxxØ type where the residues in position +1 and +2 are any amino acids, and +3 is a bulky amino acid with a hydrophobic side chain. The phosphorylation state of the Y residue in the YxxØ sequence serves as a regulatory switch that determines whether the protein is retained or removed from the plasma membrane by endocytosis [57]. Phosphorylation of the Y residue inhibits the interaction of YxxØ with the μ2 subunit of AP-2 and prevents endocytosis. By contrast, dephosphorylation of the Y residue allows the YxxØ to interact with AP-2 and facilitates endocytosis. According to this model, ß-arrestin would sequester nephrin from interacting with podocin in lipid rafts, and would prevent Fyn-mediated phosphorylation of Y^1193^ allowing nephrin to undergo CDE. ß-arrestin is also known to utilize ubiquitination as means of receptor downregulation and degradation [56]. Thus, dephosphorylation of Y^1193^ could serve as an alternative or additional mechanism of nephrin endocytosis by ubiquitination. Adding another level of complexity, ß-arrestin was reported to scaffold Src kinases [56]. In view of this finding it is difficult to reconcile how ß-arrestin would prevent phosphorylation of Y^1193^ by Fyn.

PKC-α induces nephrin endocytosis and leads to proteinuria and the loss of PKC-α prevents nephrin depletion in diabetic nephropathy [58, 59]. Interestingly, PKC-α phosphorylates T^1120^ and T^1125^ residues in the nephrin intracellular domain and facilitates nephrin interaction with ß-arrestin in murine podocytes implicating that PKC-α may regulate nephrin endocytosis [52]. Even though nephrin abundance in the plasma membrane of transiently transfected fibroblasts was increased by the dominant negative dynamin and Eps15, implicating that nephrin undergoes CDE in these cells and it was decreased by high glucose that stimulates PKC-α the specific mechanism of ß-arrestin-mediated nephrin endocytosis in podocytes remains to be determined. ß-arrestin determines postendocytic itineraries of several receptors. Following CDE, receptors are transported either to the fast or slow recycling route, depending on the stability of interaction between the receptor and ß-arrestin. Nothing is currently known about postendocytic sorting of nephrin. Understanding the kinetics of binding between nephrin and ß-arrestin may help to elucidate postendocytic itineraries of nephrin and their impact on nephrin interactions with other GSD proteins.

**Fig. 1:**
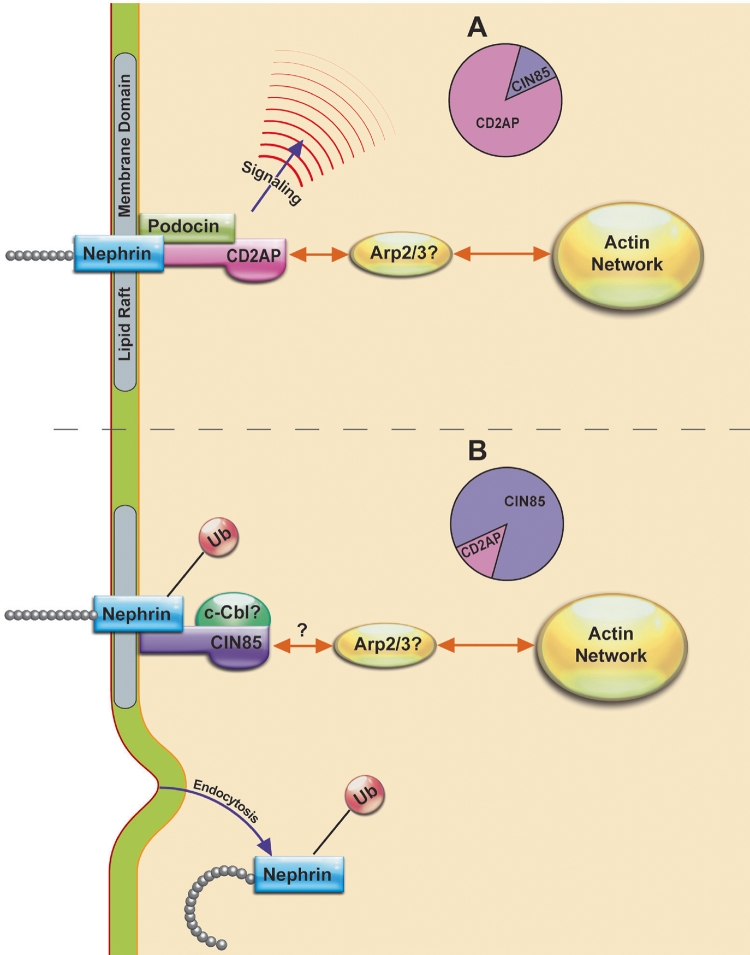
Hypothetical model for nephrin regulation by CD2AP and CIN85. Under physiological conditions, CD2AP represses CIN85 expression (**a**). CIN85 abundance increases after CD2AP depletion (**b**). Hypothetical members of the network are identified by the question mark. The double head orange arrows indicate protein–protein interactions. The single head blue arrows indicate cellular effects. Arp2/3, actin related protein 2 (ARP2) and ARP3; CD2AP, CD2-associated protein2; CIN85, Cbl-interacting protein of 85 kDa; Cbl, Casitas B-lineage Lymphoma; Ub, ubiquitin

CD2AP is another member of the GSD localized in the cytoplasmic region juxtaposed to lipid raft membrane domains. CD2AP interacts with podocin and nephrin and anchors the proteins to the actin cytoskeleton stabilizing the GSD [60]. CIN85/Ruk_L_ is a closely related homologue of CD2AP [61]. In intact podocytes CD2AP represses CIN85/Ruk_L_ expression by SUMOylation while the loss of CD2AP removes repression and results in increased CIN85/Ruk_L_ abundance and leads to CIN85/Ruk_L_-mediated nephrin ubiquitination and endocytosis [62, 63]. The model elucidating why CIN85 behaves towards nephrin in a manner opposite to CD2AP explains that unlike CD2AP, CIN85 does not contain an actin-binding domain [62, 64]. It follows that absence of CD2AP would impair nephrin partitioning to lipid rafts, increase CIN85 abundance, and facilitate nephrin interaction with CIN85. However, other investigators have shown that CIN85 directly interacts with actin and together with CD2AP bundles actin filaments and modulates podocyte migration [65]. Moreover, CIN85 clusters Src, provides a platform for actin related protein 2 (ARP2) and ARP3 (Arp2/3)-mediated actin assembly, and plays a role in cell polarization and motility (Fig. 1). The cell- and culture-dependent factors as well as expression of different CIN85 splice variants may, at least in part reconcile differences in CIN85 functions demonstrated by different investigators. The ubiquitin ligase that mediates nephrin ubiquitination has not been determined. c-Cbl is the E3 enzyme originally identified as a binding partner of CIN85 and mediates ubiquitination of many receptors [66]. It will be interesting to determine whether c-Cbl cooperates with CIN85 and ubiquitinates nephrin in podocytes. After recruiting c-Cbl to target membranes and receptors, CIN85 links the ubiquitinated receptor with endophilins A, which facilitate formation of CCPs [67]. Future studies may elucidate whether similar mechanism takes place in podocytes during nephrin endocytosis.

While Notch signaling regulates podocyte development, its activation in the mature kidney alters FP architecture and induces proteinuria by a mechanism that involves nephrin endocytosis in a dynamin-dependent and lipid raft-independent manner [53]. At present, our understanding of the mechanisms and purpose of nephrin endocytosis is incomplete. For example, current data are insufficient to propose a unified model explaining whether nephrin signaling is either attenuated or amplified or both by nephrin endocytosis. Moreover, the relative contribution of different mechanisms of nephrin endocytic remains unknown. Understanding the mechanisms, function, and regulation of nephrin endocytosis is critical for developing therapeutic interventions to stabilize nephrin at the GSD.

Cadherins, the transmembrane adhesion receptors present at the AJ of epithelial cells are important for establishing cell-cell contact during embryonic development and maturity in a variety of epithelia, including podocytes [68]. The intracellular domains of cadherin interact with a variety of proteins that indirectly link cadherins with the contractile actin cytoskeleton while the extracellular domains interact in *trans* with cadherins on adjacent cells or in *cis* with cadherins within the same membrane [69]. While the physiology of the *cis* and *trans* interactions are not well understood, it has been increasingly recognized that endocytic trafficking is critical for regulation of *trans* interactions and control of adhesion forces [70]. The endocytic trafficking of E-cadherin has been studied extensively and the trafficking routes are cell-type and condition-dependent. It would be interesting whether future studies demonstrate the role of endocytic trafficking of cadherins in regulating adhesion forces at the GSD.

### Membrane trafficking at Focal Contacts (FC)

Integrins are adhesion molecules that regulate cell attachment and migration and inside-out and outside-in signaling [71]. Integrins organize interactions between the cell and the surrounding extracellular matrix (ECM) and influence the composition of the ECM. The α- and ß-integrin subunits form heterodimers and serve as receptors for a variety of ECM molecules. Protein scaffolds organized by integrins at the cell-ECM interface include focal adhesion kinases and FERM (4.1 protein/ezrin/radixin/moezin) domain containing proteins, such as talin. Focal adhesion kinase (FAK) and talin are present at the FP-GBM interface where FCs are formed [38]. FCs contain mainly α_3_ß_1_ integrin heterodimers that serve as receptors for laminin-10/11 and the importance of integrins in podocyte physiology is illustrated by FP effacement in α_3_-deficient mice [72]. Endocytosis and recycling of integrins are recognized as important regulatory mechanisms that control their function [71]. It was shown that defined endocytic mechanisms and adaptors selectively target-specific integrin heterodimers. ß-integrins contain conserved endocytic motifs necessary for recruitment by AP-2 and/or Disabled2 (Dab2) and for internalization via CDE; although, some integrin heterodimers undergo CIE. Integrin recycling occurs via the Rab4 and/or Rab11 route. Redistribution of integrins by endocytic trafficking adjusts their membrane abundance and spatially restricts their localization to specific cellular regions, modulates Rho GTPase signaling, influences how certain receptors interact with their ligands, and controls deposition and remodeling of fibronectin in the ECM. Recent data demonstrate that FP effacement is an integrin-dependent migratory process [73]. Moreover, activation of FAK, which regulates the spatial restriction of integrins to maintain cell migrational polarity and localizes integrin signaling to the cell front, also facilitates podocyte migration and FP effacement [71, 74]. Future studies will establish the specific role and regulation of integrin trafficking in podocytes.

### Membrane trafficking at the apical complex

The apical membrane of FPs facing Bowman’s space is covered by the sialoglycoprotein containing glycocalyx that maintains a negative surface charge of the podocyte urinary surface. Podocalyxin is the most abundant member of the sialomucin family present in the apical complex and functions as an anti-adhesin. Charge repulsion depends on podocalyxin abundance in the apical plasma membrane [75]. The correct apical localization of podocalyxin depends on its interaction with the Na^+^/H^+^ exchanger regulatory factor/ezrin/radixin/moesin-binding phosphoprotein of 50 kDa (NHERF/EBP50) that links podocalyxin to the actin cytoskeleton through ezrin [76]. The interaction is mediated via the NHERF/EBP50 PSD-95/Dlg/ZO-1 (PDZ) domain and the PDZ interacting domain of podocalyxin. PDZ-mediated protein interactions promote rapid recycling of several transmembrane proteins. Thus, it is expected that NHERF/EBP50 plays a role in the dynamic regulation of the apical abundance of podocalyxin by endocytic recycling. Podocalyxin expression is first noted during the S-shaped body stage in the podocyte precursor apical membrane and the protein is known to affect localization and function of AJCs in Madin-Darby canine kidney cells [75]. The podocalyxin interaction with NHEFR/EBP50 and ezrin is disrupted in animal models of podocyte injury, and podocalyxin uncoupling from the actin cytoskeleton correlates with loss of FPs [77]. Podocalyxin expression is decreased in human glomerulopathies associated with NS [78]. Future studies are needed to elucidate the role of membrane trafficking in the dynamic regulation of podocalyxin abundance in the podocyte apical membrane.

### Membrane trafficking and cytoskeletal dynamics

**Fig. 2:**
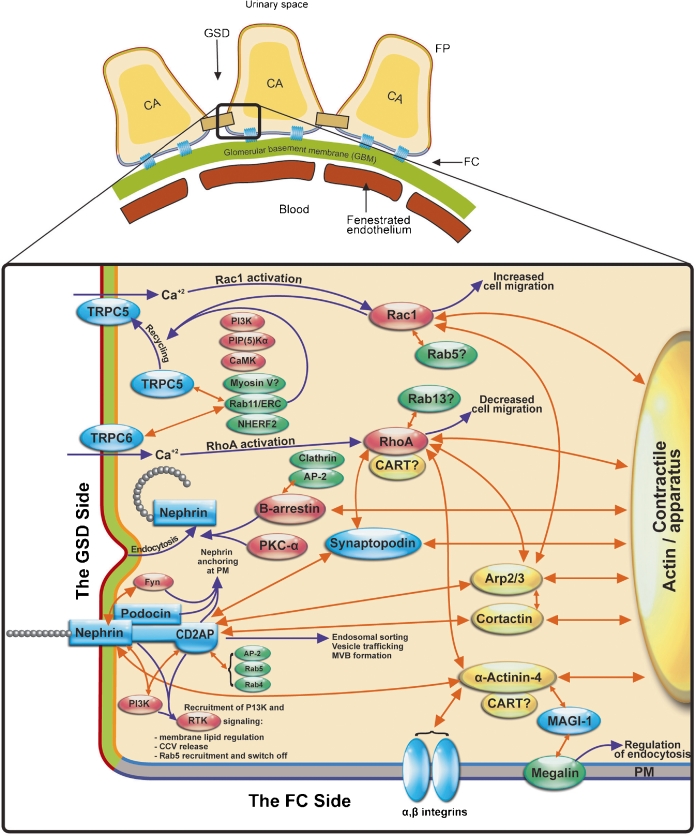
Network of physical and functional associations underlying cross-talk between membrane trafficking, cytoskeletal dynamics, and signaling in podocyte FP. Membrane bridging and juxtaposed cytoplasmic proteins (*blue*), membrane trafficking adaptors (*green*), cytoskeletal proteins (*yellow*), and signaling molecules (*red*) orchestrate membrane trafficking and intracellular signaling. Hypothetical members of the network are identified by the question mark. The double head orange arrows indicate protein-protein interactions. The single head blue arrows indicate cellular effects. AP-2, assembly polypeptide-2 complex; Arp2/3, actin related protein 2 (ARP2) and ARP3; CaMK, calcium/calmodulin-dependent protein kinase; CART, cytoskeleton associated recycling or transport complex (Hrs/α-actinin-4/myosin V); CCV, clathrin-coated vesicle; CD2AP, CD2-associated protein2; ERC, early recycling complex; MAGI, membrane-associated guanylate kinase inverted; NHERF, Na^+^/H^+^ exchanger regulatory factor; PI3K, phosphoinositide 3-OH kinase; PIP(5), phosphatidylinositol 4-phosphate 5-kinase; PKC, protein kinase C; Rab, Ras-associated binding; Rac1, Ras-related C3 botulinum toxin substrate 1; RhoA, ras homolog gene family, member A; RTK, receptor tyrosine kinase; TRPC, transient receptor potential canonical channel; CA, contractile apparatus; FC, focal contact; GSD, glomerular slit diaphragm; PM, plasma membrane

The function of mature podocytes is determined by static and dynamic properties of the cytoskeleton. Tension-bearing, vimentin-rich intermediate filaments and microtubules present in the cell body and major and secondary processes maintain cell shape and rigidity [28]. Moreover, long actin filament bundles and perimembranous, short actin networks determine the shape and function of FPs. The actin cytoskeleton connects FPs with each other via the GSD. The GSD actin network together with integral membrane protein complexes and juxtaposed cytoplasmic protein adaptors and effectors complete a highly dynamic multi-dimensional scaffold transducing signals from the extra- and intracellular environment in order to regulate glomerular filtration (Fig. 2). Actin filaments in the FP contractile apparatus change their length in response to signaling cues and regulate permeability of GFB [38]. Similar, a highly ordered scaffold of membrane proteins, actin cytoskeleton, and cytoplasmic adaptors and effectors exists at FP sole plates and regulates FCs between sole plates and the GBM [38]. During podocyte injury, actin filaments are replaced with a dense actin network and this alteration in cytoskeletal organization leads to foot process effacement [79].

There is an inseparable reciprocity between the cytoskeleton and signaling. Actin and microtubule filament networks facilitate membrane trafficking during signal transduction while cytoskeletal rearrangements and plasma membrane deformities signal to modulate endocytic trafficking. Actin regulates EE cargo sorting, membrane fission, segregation, and stretching and disturbances of the actin cytoskeleton lead to EE enlargement and sorting impairment [80, 81]. The Arp2/3 complex mediates initiation of new actin filaments and actin branching on preexisting filaments and is activated by several proteins, including cortactin. In podocytes, CD2AP colocalizes with Arp2/3 and cortactin and regulates endosomal sorting and vesicle trafficking via regulation of actin assembly [82]. Moreover, CD2AP associates with the dynamic actin pool and co-localizes with AP-2, Rab5, Rab4, and participates in formation of MVBs [82, 83]. CD2AP interacts with podocin and nephrin and thus, may function as a physical link, anchoring nephrin and podocin to the actin cytoskeleton and controlling membrane trafficking of nephrin and/or podocin through regulation of actin assembly and vesicle sorting. It is interesting whether future studies will demonstrate a direct role of CD2AP in nephrin and/or podocin membrane trafficking.

Several other factors connect actin dynamics and membrane trafficking at the GSD and FCs. α-actinin-4 cross-links actin and provides structural stability for cell-cell contact by connecting actin filaments with the cell adhesion receptors, α and ß integrins, and with the TJ and GSD protein, membrane-associated guanylate kinase inverted (MAGI)-1 [84]. At the GSD, α-actinin-4 interacts directly with nephrin and forms a multiprotein scaffold with other integral membrane and cytoplasmic proteins [38]. Direct binding between MAGI-1 and megalin—both members of the multi-protein network organized by α-actinin-4 was proposed to play a role in network assembly in podocytes. Megalin is a member of the LDL receptor family and an endocytic adaptor and facilitates endocytosis of exogenous α-galactosidase A in podocytes from patients with Fabry disease [85]. These data demonstrate that megalin may facilitate endocytosis in podocytes. As a member of the CART (cytoskeleton-associated recycling or transport): an Hrs/actinin-4/BERP/myosin V, α-actinin-4 mediates constitutive recycling of the transmembrane receptors via the rapid recycling route in an actin-dependent manner [86]. Practically nothing is known about the role of α-actinin-4 or other members of the CART complex in trafficking of multi-protein scaffolds at the GSD or FCs. α-actinin-4 gene mutations are associated with focal segmental glomerulosclerosis (FSGS) [87]. Even though the mechanisms are far from being understood, cytoplasmic sequestration of transcription factors caused by α-actinin-4 gene mutations suggests that α-actinin-4 plays an important role in membrane trafficking [87]. Future studies will determine whether α-actinin-4 controls transcriptional responses in podocytes via actin-dependent membrane trafficking events.

The Rho family of small GTPases: Ras homolog gene family, member A (RhoA), Cdc42, and Ras-related C3 botulinum toxin substrate 1 (Rac1) regulate many aspects of physiological and pathological cell behaviors, including actin dynamics and cell motility and migration [88]. For example, rapid cytoskeletal rearrangements mediated by actin assembly and actin filament length changes in response to external mechanical stress are mediated by Rho signaling. In podocytes, RhoA signaling is regulated by synaptopodin, a member of the multi-protein scaffold at the interface between FP sole plates and the GBM and leads to formation of actin stress fibers [89]. Nothing is currently known about the regulation of RhoA trafficking in podocytes. In endothelial cells of sprouting vessels, RhoA translocates from the cell-cell junction to the cell leading edge in Rab13-positive endocytic vesicles containing several factors necessary for Rho activation and downstream signaling, including α-actinin-4 [90]. Rab13 also facilitates assembly of the TJ and cell-cell junctions suggesting that junctional proteins may play a role in cell migration [91]. Similar to RhoA, activation and trafficking of Rac1 occurs in Rab5-positive endocytic vesicles and is necessary for Rac1 signaling [92]. Whether RhoA signaling is regulated by membrane trafficking in podocytes remains to be determined.

Rho GTPases are activated by calcium (Ca^+2^) influx generated by transient receptor potential (TRP) canonical channels in cellular microdomains associated with recruitment of either RhoA or Rac1 [93, 94]. In podocytes and fibroblasts, TRPC5-mediated Ca^+2^ influx activates Rac1 and promotes cell migration [95]. By contrast, TRPC6-mediated Ca^+2^ influx activates RhoA and inhibits cell migration. Ca^+2^ ions are critically important for signaling in podocytes by a variety of mechanisms and mutations in the *TRPC6* gene are associated with FSGS [94]. Adjusting channel abundance at the plasma membrane—because these channels are constitutively active at physiological membrane potentials—regulates Ca^+2^ influx [93, 96, 97]. Other mechanisms, including myosin light chain kinase and calcium/calmodulin-dependent protein kinase (CaMK) are also involved in the channel activation process and may be cell-type-specific [98, 99]. Membrane trafficking is critical for Ca^+2^ influx in cells, such as podocytes where the influx depends on the plasma membrane abundance of TRPC5 and TRPC6. In fibroblasts, translocation of active TRPC5 from a submembranous compartment to the plasma membrane is induced by EGF and requires Rac1, PI3K, and phosphatidylinositol 4-phosphate 5-kinase alpha (PIP(5)Kα) [100]. Channel translocation increases Ca^+2^ influx and initiates morphological changes in cells in response to stimuli. Translocation of TRPC5 and/or TRPC6 to the plasma membrane likely occurs in Rab11-positive recycling vesicles associated with ERC and requires NHERF2 [101]. TRPC6 localizes to the GSD and together with podocin and the juxtaposed actin cytoskeleton is hypothesized to function as a sensor of osmotically and mechanically induced membrane stretch [102]. Myosin V is a progressive, non-conventional motor and a mechanosensor that transports cargo in Rab11-positive recycling vesicles on actin filaments to the plasma membrane [103]. The myosin V motor activity requires CaMK and a tight control of Ca^+2^ concentration in microdomains referred to as regulated motor units [104, 105]. Based on the information, it is reasonable to predict that myosin V may transport TRPC5 and/or TRPC6 in Rab11-positive recycling vesicles and that the channels may regulate myosin V function by controlling Ca^+2^ influx in myosin V-based-regulated motor units. Future studies will need to examine the prediction.

Nephrin and CD2AP interact with the p85 regulatory subunit of the phosphoinositide 3-OH kinase (PI3K) and recruit PI3K to the plasma membrane [106]. PI3K plays an essential role in a multi-level regulation of receptor tyrosine kinases (RTK) trafficking, including regulation of membrane lipid composition, release of CCVs, and the Rab5 recruitment and switch off [107]. These functions are critical to RTK signaling. The interactions of PI3K with nephrin, CD2AP, and several other GSD protein networks indicate an important role of PI3K regulated membrane trafficking in several aspects of GSD function. For example, a recent study demonstrated that nephrin mediates actin reorganization in podocytes via PI3K [108]. More studies examining cross-talk between actin dynamics and membrane trafficking at the GDS may elucidate how cytoskeletal remodeling affects membrane trafficking and signaling in podocytes and how mutations in proteins controlling cross-talk lead to cytoskeletal rearrangements, FP effacement, and proteinuria.

### Membrane trafficking and podocyte injury

The pervasiveness of membrane trafficking in virtually every event of cell regulation predicts that alterations of endocytic machinery and intracellular sorting should play an important role in human pathology at large. Indeed, the pathogenesis of many diseases can be traced back to disrupted membrane trafficking [1]. Below is a brief review of studies, not included in the previous sections that illustrate the impact of abnormal membrane trafficking on podocyte injury and glomerular disease. Plasma obtained from patients with NS reversibly displaces nephrin, podocin, and CD2AP from the cell surface into the cytoplasm in cultured human podocytes [109]. While nephrotic plasma may be deficient in factor(s) critical for cell polarization and podocyte function, as demonstrated by the effects of the podocyte-specific loss of Cdc42 [31, 32], it is also plausible that nephrotic plasma may contain abnormal factor(s) that alter membrane trafficking in podocytes. Such factor(s) could be generated by abnormal cell signaling in injured podocytes, increased mechanical stress (hyperfiltration-mediated injury), or abnormal podocyte adhesion forces [110, 111]. Examples of factors likely affecting membrane trafficking in podocytes are discussed below.

Membrane trafficking is critically involved in the execution of epithelial-mesenchymal transformation (EMT) that accompanies glomerular fibrosis, including TGF-ß induced EMT in diabetic nephropathy [112]. Moreover, certain mutations in the *TRPC6* gene affect channel trafficking and prevent its insertion in the GSD membrane leading to abnormal Ca^+2^ homeostasis and signaling, podocyte injury and FSGS [113]. By contrast, activation of wild type TRPC6 or activating mutations in the channel also lead to podocyte injury and proteinuria indicating that control of TRPC6 trafficking and cell membrane abundance are critical for podocyte function [114]. Parenthetically, adjusting channel abundance in the GSD by regulating its endocytic trafficking may serve as a potential therapeutic approach to normalize TRPC6 signaling. Whether inflammatory mediators affect GSD integrity by modulating endocytosis and recycling of junctional proteins, as documented in intestinal epithelial cells, remains unknown [115].

Renin-angiotensin system (RAS) activation, as seen in glomerular hyperfiltration is an important cause of podocyte injury and proteinuria [116]. In persistent RAS activation, angiotensin II leads to rearrangement of the actin cytoskeleton resulting in a podocyte phenotype switch to migratory by mechanisms involving activation of Rac-1 and FERM proteins, downregulation of 〈-actinin-4, and reduction of FC number [117]. Moreover, angiotensin II decreases abundance of GSD proteins and induces nephrin dephosphorylation by the caveolin-dependent mechanism, suggesting a role of CIE in this process [118]. The synthetic erythropoietin, darbepoetin protects podocytes from injury by a mechanism that involves restoration of nephrin expression in the GSD [119]. It would be interesting to determine whether darbepoetin regulates membrane trafficking of nephrin or other GSD proteins. Elevated levels of serum soluble urokinase receptor (suPAR), observed in patients with FSGS activates ß_3_ integrin and lead to FP effacement, glomerulopathy, and proteinuria [120]. It can be predicted that podocyte injury caused by suPAR via ß_3_ integrin activation involves altered podocyte adhesion to the GBM mediated by altered membrane trafficking.

## Conclusions and perspectives

Membrane trafficking is the master regulator of virtually every cellular function. Although little evidence exists, studies in other organ systems strongly indicate that the versatility of membrane trafficking allows dynamic regulation of elaborate protein scaffolds and signaling cascades during podocyte development, differentiation, and maintenance of manure podocytes. Moreover altered membrane trafficking may be the cause and/or the result of podocyte injury. Proteinuria is an important health problem associated with diseases affecting the GFB and diagnostic tools and treatment options are limited and not optimal at best. Biomedical and pharmaceutical applications of nanotechnology exploiting endocytosis and transcytosis for targeted delivery of diagnostics and therapeutics have been gaining momentum in other medical fields [121]. Cultured podocytes internalize proteins via endocytosis and this trafficking route has been explored as a method for drug delivery to injured podocytes [122, 123]. Future studies revealing the role, mechanisms, and regulation of membrane trafficking in podocyte health and disease may help to design diagnostic approaches and targeted treatments for proteinuria. Since protein trafficking is cell- and polarization-dependent, one of the future challenges would be to study membrane trafficking in human podocytes.
